# Residue Analysis and the Effect of Preharvest Forchlorfenuron (CPPU) Application on On-Tree Quality Maintenance of Ripe Fruit in “Feizixiao” Litchi (*Litchi chinensis* Sonn.)

**DOI:** 10.3389/fpls.2022.829635

**Published:** 2022-03-04

**Authors:** Xin-Sheng Liu, Ye-Cheng Luo, Si-Wei Wang, Hui-Cong Wang, Smadar Harpaz-Saad, Xu-Ming Huang

**Affiliations:** ^1^Guangdong Litchi Engineering Research Center, College of Horticulture, South China Agricultural University, Guangzhou, China; ^2^Plant Protection Research Institute, Guangdong Academy of Agricultural Science, Guangzhou, China; ^3^The Robert H. Smith Institute of Plant Sciences and Genetics in Agriculture, Faculty of Agriculture, Hebrew University of Jerusalem, Rehovot, Israel

**Keywords:** litchi, cytokinin, fruit ripening, anaerobic respiration, hanging life, quality

## Abstract

Litchi is a highly perishable fruit. Ripe litchi fruit loses quality quickly as they hang on tree, giving a very short hanging life and thus harvest period. This study attempted to explore the roles of cytokinin in regulating fruit ripening and senescence of litchi and examine the possibility of applying cytokinin in “on-tree storage” of the fruit. Exogenous cytokinin, forchlorfenuron (CPPU), was applied at 20 mg L^−1^ 7 weeks after full bloom on litchi (*Litchi chinensis* cv. Feizixiao) fruit clusters. Color parameters, chlorophylls, anthocyanins, fruit and fruit part weights, total soluble solutes (TSSs), soluble sugars, organic acids, non-anthocyanin flavonoids, ethanol, and also CPPU residue in fruit were traced. CPPU residue was higher but decreased faster in the pericarp than in the aril, where it maintained < 10 μg kg^−1^. CPPU had no significant effect on fruit weight but tended to increase pericarp weight. The treatment suppressed chlorophyll loss and anthocyanin accumulation in the pericarp, increased non-anthocyanin flavonoids in the aril, but had no significant effects on non-anthocyanin flavonoids in the pericarp and total sugar and organic acids in the aril. As the commercially ripe fruit hanged on tree, TSSs, total sugar, and sucrose decreased with ethanol and acetic acid accumulation in the aril. CPPU significantly suppressed the loss of sucrose and total sugar and the accumulation of ethanol and acetic acid in the aril and inhibited malondialdehyde accumulation in the pericarp of the overripe fruit. Soluble invertase, alcohol dehydrogenase, and pyruvate decarboxylase (PDC) activity and gene expression in the aril were downregulated by CPPU. The results suggest that cytokinin partially suppresses the ripening process in litchi and is effective to slow quality loss in the overripe fruit on tree.

## Introduction

Fruit ripening occurs with an orchestrated array of biological events such as loss of chlorophylls and acids, accumulation of pigments and sugars, emission of aromatic substances, and changes in tissue texture, which is known as “ripening syndrome.” Fruit ripening can also be considered as an early phase of fruit senescence with the loss of chlorophylls and the breakdown of cell membrane integrity resembling leaf senescence (Huang and Wu, 2006). The progress of senescence in ripe fruit determines its “hanging life,” which differs greatly among species. Litchi (*Litchi chinensis* Sonn.), a tropical fruit crop native to south China, produces a highly perishable fruit with a very short shelf-life. Detached fruit is prone to browning chiefly due to desiccation that induces cell breakdown (Underhill and Simons, 1993; Huang et al., 2001, 2005). However, on-tree litchi is also prone to quality loss as it becomes overripe. Overripe litchi fruit loses sugars (Wang et al., 2003) and develops off-flavor with the accumulation of alcohol and acetaldehyde that reduces fruit storability (Pesis et al., 2002; Huang et al., 2005; Huang and Wu, 2006). The short hanging life (7–10 days) of litchi provides very little flexibility in harvesting time and creates a heavy burden in the marketing of the ripe fruit in major producing countries such as China, India, and Vietnam (Huang, 2020).

Cytokinins are well-known for their effect in suppressing leaf senescence (Robson et al., 2004). They are also generally considered as a suppressor of fruit ripening (NeSmith, 2002; Wang et al., 2007; Peppi and Fidelibus, 2008). Reduction in active cytokinins and gibberellins is considered as a precondition for the occurrence of fruit ripening in loquat (Reig et al., 2016). In grapes, exogenous cytokinin treatment with forchlorfenuron, N-(2-chloro-4-pyridyl), N′-phenylurea (CPPU), has been shown to increase fruit weight and suppress ripening events, such as fruit coloration, softening, soluble solid accumulation, and tannin decomposition (Peppi and Fidelibus, 2008; Maoz et al., 2014). CPPU is effective in enlarging fruit size or weight and delaying fruit ripening in blueberry (NeSmith, 2002). In kiwifruit, however, CPPU advances fruit ripening events such as fruit softening, sugar accumulation, and flesh coloration (Lötter, 1992). In litchi, CPPU effectively delays fruit coloration (Stern et al., 2006; Fahima et al., 2019) as a result of downregulation of anthocyanin biosynthesis and chlorophyll degradation genes alongside upregulation of chlorophyll biosynthesis gene expression (Wei et al., 2011; Lai et al., 2015). However, 5–20 mg L^−1^ CPPU treatment did not significantly affect soluble solutes, acidity, and fruit weight but extended the hanging life of “Mauritius” by 2–3 weeks and enhanced postharvest performance (Stern et al., 2006; Fahima et al., 2019). Transfusion of 10 mg L^−1^ zeatin to detached fruit cluster of litchi slowed membrane leakage increase (Huang et al., 2001). However, beyond the effects on pigment levels in litchi fruit pericarp, there is little information about on-tree changes in internal quality traits in CPPU-treated fruit. We hypothesized that this senescence-suppressing hormone regulates part of the ripening process and is effective to slow quality loss as it delays the senescence of litchi fruit.

To test this hypothesis, this study examined the effect of 20 mg L^−1^ CPPU treatment on the on-tree changes in quality-related components in litchi fruit after they reached commercial maturity. The main goal of this study was to obtain an understanding of the roles of cytokinin in the regulation of fruit ripening and on-tree senescence of litchi. Additionally, the study also aimed to examine the possibility of using CPPU in “on-tree storage” of litchi, which might be practical to reduce the burden during the short harvesting and marketing season of the fruit.

In practice, CPPU is generally applied at very low concentration due to its strong cytokinin activity, and residue of CPPU in the fruit flesh is generally below maximum residue limit (MRL; Chen et al., 2013; Li et al., 2020). However, the potential health risk of CPPU is still under evaluation (Bu et al., 2019). Therefore, in this study, residues of CPPU in the pericarp and aril tissues were analyzed following application to provide reference about food safety related to preharvest application of CPPU.

## Materials and Methods

### Materials and Treatments

The experiment was carried out over the seasons of 2018–2019 in a commercial orchard located in Yangxi, west of Guangdong Province, China, with 20-year-old trees of cv. “Feizixiao,” the most widely grown and major exported cultivar in China. The trees were under routine managements for fertilization, irrigation, pests, and diseases.

According to result of Stern et al. (2006), CPPU treatment in this experiment was conducted at 7 weeks after full bloom (WAFB), that is, about 3 weeks before harvest. Our preliminary experiment showed that CPPU treatments at 2.5, 5, 10, and 20 mg L^−1^ 7 WAFB had no significant effect on total soluble solute (TSS) accumulation in ripe fruit of “Feizixiao” but tended to maintain a higher TSS in overripe fruit at a higher concentration (Supplementary Figure 1). Therefore, CPPU treatment at 20 mg L^−1^ was used in this experiment. Five trees with similar loads and at the same phenological status were selected and tagged. Each tree served as an experimental block, where 16 panicles (fruit clusters) each with 16–20 fruit were selected from all directions of the canopy and evenly divided into two groups each with 8 panicles. In one group, the 8 panicles were sprayed with clean water added with 0.01% (v/v) Tween-20 till drip-off, which served as the control; in the other group, the panicles were sprayed with 20 mg L^−1^ CPPU with 0.01% (v/v) Tween-20 until drip-off. One fruit was collected weekly from each panicle until 3 weeks after commercial ripeness was attained. The 8 fruits from the same tree in the same treatment were pooled as one replicate sample. The sampled fruit was individually measured for fruit weight with an electronic balance at an accuracy of 0.01 g (MP1002, SOPTOP, Shanghai, China) and color parameters (*a* value for redness and *b* value for yellowness) with a chromameter (CR-400, Minolta, Japan). The fruit was then dissected into the aril and the pericarp. After the fresh weight of each tissue was obtained with an electronic balance at an accuracy of 0.01 g and TSS in the aril determined with a PAL-1 digital refractometer (Atago, Japan), the aril and the pericarp were separately frozen, ground into powder in liquid nitrogen, and stored in a freezer at −80°C for analyses of components, enzymatic activity, and gene expression.

### CPPU Residue Analysis

Samples for CPPU residue analysis were taken at 0 (2 h after spray), 2, and 4 weeks after CPPU application (WACA). A total of 2.0 g of pericarp or aril tissue powder was transferred into a centrifuge tube containing 10 mL acetonitrile and vortexed for 30 min. A total of 1 g of sodium chloride was added to the mixture and vortexed for 5 min before centrifuged at 5,000 g for 5 min. The supernatant acetonitrile phase was collected and forced through 0.22-μm microfiltration membrane. The filtrate was ready for HPLC-MS/MS analysis. The instrument used was an Agilent LC-1200 HPLC (Agilent Technologies Co., Ltd., USA) coupled with a triple quadrupole tandem mass spectrometer [AB Sciex 4000Q Trap (AB SCIEX, USA)] equipped with an ESI ion source. The HPLC working conditions included an Agilent poroshell EC C18 column (150 mm × 3.0 mm, 2.7 μm) (Agilent Technologies Co., Ltd., USA) at 35°C, a mobile phase of acetonitrile: 0.1% formic acid aquatic solution (4:1) at a flow rate of 0.50 mL/min and a sample injection volume of 10 μL. The ion source parameters included an ion spray voltage of 5,500 V and a source temperature of 550°C. Multiple reaction monitor was used for detecting CPPU by monitoring ion transitions of m/z 248.0/129.0 and m/z 248.0/93.0. Analytical pure CPPU was used to prepare a series of gradient solutions in the range of 1–500 μg L^−1^, which were used as the internal standard for construction of the standard curve separately for the pericarp and the aril. Both showed a perfect linear function (*R*^2^ > 0.999, *p* < 0.001) between CPPU concentration and peak area.

### Sugar Analysis

Sugar extraction from the aril and analysis with HPLC were carried out according to Yang et al. (2013). Briefly, enzymes in the aril samples each of 0.1 g were denatured in a water bath at 100°C for 2 min before they were homogenized in 10 mL distilled water. A total of 2 mL of the homogenate was centrifuged at 13,000 g for 10 min at room temperature. A total of 1 mL of the supernatant was forced through a Sep-Pak®1cc (100 mg) C18 cartridge (Waters, USA). The filtrate was ready for HPLC analysis for sugars. An Agilent 1200 HPLC system (Agilent Technologies Co., Ltd., USA) installed with a Transgenomic CARB Sep Coregel 87C column (CHO-99-5860) (ANPEL Lab Technologies, Shanghai, China) and a guard Transgenomic CARB Sep Coregel 87C cartridge (ANPEL Lab Technologies, Shanghai, China) maintained at 80°C. The detector was a G1362A refractive index detector (Agilent Technologies Co., Ltd., USA), which operated at 40°C. The injection volume was 10 μL and the mobile phase was double distilled water with a flow rate of 0.6 mL min^−1^. Quantification of sugars (sucrose, fructose, and glucose) in the samples was performed according to external standard curves constructed using standard sugar solutions at 0.625, 1.25, 2.5, 5.0, and 10.0 mg mL^−1^.

### Anthocyanin and Chlorophyll Measurement

Anthocyanin content in the pericarp was measured using the pH differential method of Fuleki and Francis (1968). Pericarp powder of known weight (0.5 g) was suspended in 10 mL of 0.1 m HCl solution and allowed to stand for 5 h. An amount of 1 mL from the upper clear extract was pipetted into a test tube and diluted with 5 mL of 0.4 m KCl–HCl buffer (pH 1.0) or with 5 mL of 0.4 m citric acid–Na_2_HPO_4_ buffer (pH 4.5). The optical density at 520 nm (OD520) of the dilutions was measured with a UV2550 spectrophotometer (Shimadzu, Kyoto, Japan). The difference in OD520 between pH 1.0 and pH 4.5 of the same extraction was used to calculate total anthocyanin concentration using a standard curve constructed with cyanidin-3-glucoside solutions at 62.5, 125, 250, and 500 mg L^−1^.

Chlorophylls in the pericarp were extracted in 80% acetone for 24 h in darkness, and the OD645 and OD663 of the extracts were measured with a UV2550 spectrophotometer (Shimadzu, Kyoto, Japan). Contents of chlorophyll a, chlorophyll b, and total chlorophylls were calculated according to Arnon (1949).

### Organic Acid Analysis

Aril powder of known weight (0.2 g) was homogenized in 2 mL 0.2% (w/v) HPO_3_, and the homogenate was centrifuged at 10,000 g for 15 min at 4°C. The sediment was suspended with 2 mL 0.2% HPO_3_ and centrifuged at 10,000 g for 15 min. The combined supernatant was brought to 5 mL. An amount of 1 mL of this extract was filtered through a 0.45-μm Millipore TM filter, and the filtrate was then analyzed with an Agilent 1100 HPLC (Agilent Technologies Co., Ltd., USA) equipped with a NUCLEODUR C18 column (250 mm × 4.6 mm) (Pretech Instruments, Sollentuna, Sweden) set at 35°C, an Agilent G1311A pump, an automated injector, and an Agilent G1314A UV–spectrophotometer (Agilent Technologies Co., Ltd., USA). Detection of organic acids was performed at 210 nm using a diode array detector. The mobile phase was 0.2% (w/v) HPO_3_ solution at a flow rate of 1 mL min^−1^. Analytical pure ascorbic, malic, tartaric acid, and acetic acid were used to construct the standard curves of peak areas vs. concentrations (62.5, 125, 250, 500, and 1,000 μg/mL for malic acid and tartaric acid; 12.5, 25, 50, 100, and 200 μg mL^−1^ for ascorbic acid). The regressed linear equations were used to quantify the contents of the above organic acids in the tissue.

### Flavonoids in the Pericarp and the Aril

Aril or pericarp tissue (0.5 mg) was ground into homogenate in 2.0 mL of 70% methanol containing 2% formic acid at 4°C. After being shaken at 30°C for 30 min in a thermomixer at 1,000 g, the homogenate was centrifuged at 10,000 g for 10 min. The supernatant was forced through a 0.45-mL syringe filter prior to HPLC analysis with an Agilent 1200 HPLC equipped with a diode array detector (Agilent Technologies Co., Ltd., USA), and an Inertsil ODS-3 column (5.0 μm particle size, 4.6 mm × 250 mm) (GL Sciences Inc., Tokyo, Japan) was used in the separation, preceded by an Inertsil ODS-3 Guard Column (5.0 μm, 4.0 mm × 10 mm) (GL Sciences Inc., Tokyo, Japan) maintained at 30°C. A total of 10 μL of the filtered supernatant was injected to HPLC. Two solvents were used for the separation in a gradient system with a flow rate of 1.0 mL min^−1^. Solvent A consisted of 10% formic acid in water, and solvent B consisted of 10% formic acid and 1.36% water in acetonitrile. The gradient was 95% A (0 min), 85% A (25 min), 78% A (42 min), 64% A (60 min), and 95% A (65 min). Postrun time was 10 min. Flavonoid monitoring was performed at 280 nm. Peaks were identified by the comparison of retention time and UV spectra with corresponding authentic standards. The concentration of individual phenolic compounds was determined based on peak area and calibration curves derived from corresponding authentic phenolic compounds (62.5, 125, 250, and 500 μg mL^−1^). The phenolic standards were obtained from Sigma-Aldrich (St. Louis, MO, USA), Extrasynthese (Genay Cedex, France), or AApin Chemicals (Abingdon, Oxon, UK).

### Determination of Ethanol, Malondialdehyde, and Acetic Acid in the Aril

Ethanol was measured using potassium dichromate colorimetric method according to Ge et al. (2011). A total of 1 g of aril powder was added to 0.5 mL saturated sodium chloride solution in a 10-mL vial, to which 2 mL of potassium dichromate sulfate solution (0.2 g potassium dichromate dissolved in 100 mL of 9 M sulfate solution). The vial was then sealed with rubber cap and duct tape and placed in an oven set at 55°C for 2 h. After the vial cooled down to room temperature, it was shaken violently to allow ethanol in the tissue to be oxidized thoroughly. Then, 8 mL of pure water was added into the vial, and the OD600 of the liquid in the vial was collected with a UV2550 spectrophotometer (Shimadzu, Kyoto, Japan) and used to quantified ethanol content.

For extraction and determination of malondialdehyde (MDA), 1 g of aril powder was homogenized in 5 mL of 100 g L^−1^ trichloroacetic acid solution at 4°C. The homogenate was centrifuged at 10,000 g for 20 min. Two milliliters of the supernatant was added to a test tube containing 2 mL of 0.67% thiobarbituric acid solution. The mixture was bathed in boiling water for 20 min. After cooling down, optical density at 450, 532, and 600 nm was measured with a UV2550 spectrophotometer (Shimadzu, Kyoto, Japan). MDA concentration (C) in the reaction mixture was calculated according to the following equation:


$$\begin{array}{l} C\left(\text{μ}molL^{-1}\right)=6.45\times \left(OD_{532}-OD_{600}\right)-0.56\times OD_{450} \end{array}$$

MDA content on tissue basis (mmol kg^−1^) was calculated according the following equation:

MDA content (mmol kg^−1^) = C × V/VS/m

where V stands for extraction volume, Vs for volume added to the reaction mixture, and m for the amount of tissue used for the extraction.

### Expression of Genes Related to Sucrose Hydrolysis and Anaerobic Respiration

Since our result showed that sucrose and total sugar content in the aril maintained significantly higher, whereas anaerobic respiration products, for example, ethanol and acetic acid, were significantly lower following CPPU treatment, we analyzed the expression of genes encoding key enzymes involved in anaerobic respiration, for example, pyruvate decarboxylase (PDC) and alcohol dehydrogenase (ADH), and in sucrose hydrolysis (neutral and acid invertases). Genes annotated to PDC (*LcPDC1* and *LcPDC2*), ADH (*LcADH1* and *LcADHL1*), soluble neutral invertase (SNIV) (*LcSNIV1, LcSNIV2, LcSNIV3*, and *LsSNIV4*), and soluble acid invertase (SAIV) (*LcSAIV1* and *LcSAIV2*), which were differentially expressed between CPPU treatment and the control based on our RNA-seq data (unpublished), were selected for qPCR analysis, which was conducted with 3 technical replicates. Their identity and description were deposited in the litchi genome database (http://111.230.180.7:84/), and the sequences of primer used for qPCR analysis are shown in Supplementary Table 1.

### Assays of Soluble Invertases, ADH, and PDC in the Aril

Crude enzyme extracts were prepared following the procedures described by Yang et al. (2013). Aril powder (1.0 g) was homogenized in 1.5 mL of extraction buffer containing 100 mM Hepes–NaOH (pH 7.5), 5 mM MgCl_2_, 1 mM EDTA, 2.5 mM DDT, 1% (v/v) Triton X-100, 5% (w/v) PVPP, 0.5% (w/v) BSA, and 10% (v/v) glycerol. The extract was centrifuged at 13,000 g for 10 min in an Eppendorf microcentrifuge. The supernatant (1 mL) was desalted with a PD-10 column equilibrated with 25 mM Hepes–NaOH (pH 7.5) containing 30% glycerol, 5 mM MgCl_2_, and 1 mM EDTA to a final volume of 2 mL. The eluate collected was used as the crude enzyme. The activities of (EC. 3.2.1.26) SAIV and SNIV (EC.3.2.1.26) were quantified by the rate of reducing sugar generation from sucrose degradation according to Gordon (1991). Then, 1 mL of reaction mixture used to determine SAIV was composed of 100 mM citrate buffer (pH 5.5), 1% sucrose, and 200 μL of desalted extract. Reaction mixtures were incubated at 34°C for 1 h, and the reaction was terminated with boiling water bath for 5 min. For the determination of SNIV activity, the reaction mixture was identical to that for SAIV except that the citrate buffer was replaced by 100 mM Hepes–NaOH (pH 7.5).

Methylbenzothiazolone hydrazone (MBTH) method was used for ADH determination. The reaction solution contained 10 mM glycine–KOH (pH 9.0), 800 μM NAD^+^, and 300 mM ethanol. The reaction was initiated by adding 20 μL of crude enzyme into 180 μL reaction solution. Thirty minutes later, 0.4 mL of 0.4% (w/v) MBTH hydrochloride hydrate solution was added to stop the reaction. The mixture was allowed to stand for 20 min and then added with 0.4 mL 0.73% (w/v) FeCl_3_ solution and fully mixed. Ten min later, 1 ml of pure water was added to the mixture and OD610 was read. Acetaldehyde solutions at gradient concentrations of 26, 31, 36, 41, and 46 μg mL^−1^ were prepared to construct a standard curve to quantify the concentration of acetaldehyde generated from the enzymic reaction. The enzyme activity in the tissue was calculated according to the equation:


$$\begin{array}{l} ADH\left(mmolkg^{-1}s^{-1}\right)=X\times V\times Vs/\left(44.05\times t\right)/Ve, \end{array}$$

where X stands for acetaldehyde concentration at the end of enzyme reaction (μg mL^−1^), V for crude enzyme volume (1.5 mL) extracted 1 g of tissue sample, Vs for reaction mixture volume (0.2 mL), t for reaction duration (30 min), Ve for volume of crude enzyme in reaction mixture (0.02 mL), and 44.05 is the molecular weight of acetaldehyde.

Pyruvic decarboxylase, which catalyzes the production of NADH and acetaldehyde from pyruvic acid, was assayed with an enzyme-linked method. In this method, acetaldehyde generated by PDC was immediately reduced by exogenous ADH with the oxidation of NADH, which was traced with the reduction in OD340. A total of 2.7 mL of 200 mM citrate buffer solution (pH 6.0) was pipetted into a colorimeter cup and injected with 0.1 mL of 1 M sodium pyruvate, 0.05 ml of 6.4 mM NADH, and 0.05 mL of 200 U mL^−1^ ADH. After fully mixed with a pipette and OD340 of the mixture became stabilized, 0.1 mL of crude enzyme was pipetted into the mixture and measured the initial OD340. Five min later, OD340 was read again. PDC enzyme activity was calculated according to the equation:


$$\begin{array}{l} PDC\left(\text{μ}molkg^{-1}s^{-1}\right)=\left(\Delta OD340/s\right)\times 3\times V/\left(6.22\times 0.1\right), \end{array}$$

where ΔOD340/s means a decrease in OD340 per second, 3 is the total volume of the reaction mixture, V stands for crude enzyme volume extracted from 1 kg of tissue sample, 6.22 is micromolar extinction coefficient of NADH at 340 nm, and 0.1 is the volume (mL) of crude enzyme added to the reaction mixture.

### Statistics

The above analyses were carried out with 5 biological replicates from 5 tree-based blocks. Sample from each tree in each treatment consisted of 8 pooled fruits at each sampling time. Differences in means between the control and the CPPU treatment were analyzed with a pairwise Student's *t*-test which was performed using SPSS 19.0.

## Results

### Residue Analysis

Dynamics of residue of sprayed CPPU on the fruit surface in the pericarp and the aril is shown in Table 1. Fruit samples taken 2 h after spraying with 20 mg mL^−1^ CPPU (0 WACA) had the highest CPPU concentration in both the pericarp and the aril. CPPU concentration in the pericarp, 1,096.7 μg kg^−1^, was over 100-folds that in the aril, 9.93 μg kg^−1^. The concentration decreased significantly with time in both tissues. However, the decrease in the pericarp was more drastic than in the aril. By 2 WACA, concentration in the pericarp had decreased by nearly 10-folds from 1,096.7 to 110.3 μg kg^−1^, whereas tissue weight increased by about 1-fold. Therefore, the decrease in CPPU concentration in the pericarp could not simply be attributed to the dilution effect due to tissue growth. The total amount of CPPU in the pericarp decreased by 4-folds, which indicates massive degradation of CPPU. In contrast, tissue weight of the aril increased by about 6-folds from 0.79 to 4.88 g within 2 weeks, whereas CPPU residue concentration decreased by only one-third from 9.93 to 6.90 μg kg^−1^. The total amount of CPPU residue in the aril increased from 7.82 to 33.71 ng per fruit. By 4 WACA, when fruit was ready for harvesting, CPPU concentration in the pericarp and the aril had dropped to 49.0 and 5.25 μg kg^−1^, respectively. The total CPPU residue in the whole fruit decreased constantly, and the decrease rate in the first 2 weeks (689.45 ng per week) was much greater than the second 2 weeks (57.2 ng per week).

**Table 1:** Tissue weight and CPPU residue in the pericarp and the aril at 2 and 4 weeks after CPPU application (WACA).

**Weeks after CPPU application**		**0**	**2**	**4**
Pericarp	Tissue weight (g)	1.64 ± 0.11c	3.58 ± 0.10b	5.06 ± 0.23a
	Concentration (μg kg^−1^)	1096.7 ± 20.48a	110.3 ± 3.48b	49.0 ± 1.22c
	Amount (ng per fruit)	1799.4 ± 33.6a	394.6 ± 11.57b	247.9 ± 11.16c
Aril	Tissue weight (g)	0.79 ± 0.10c	4.88 ± 0.50b	12.60 ± 1.00a
	Concentration (μg kg^−1^)	9.93 ± 0.09a	6.90 ± 0.11b	5.25 ± 0.24c
	Amount (ng per fruit)	7.82 ± 0.07c	33.71 ± 3.46b	66.15 ± 5.28a
Total amount (pericarp + aril) (ng per fruit)		1807.2 ± 119.8a	428.3 ± 14.0b	314.1 ± 15.2c

*Different letters following the means ± standard error (n = 5) indicate significant difference between times of sampling at p < 0.05, Duncan's multiple range test*.

### Effects on Coloration, Fruit Weight, and TSS

**Figure 1:**
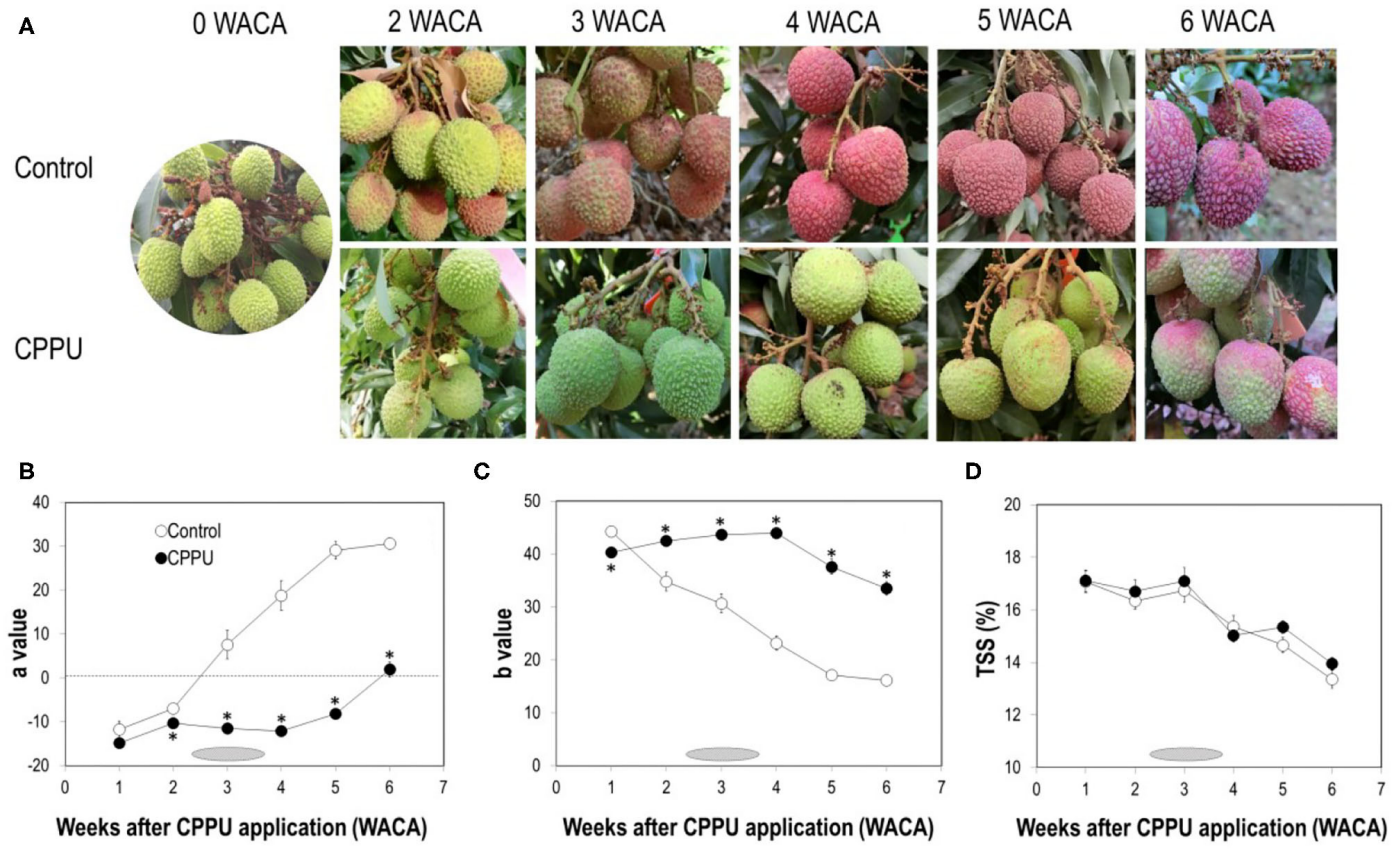
Effect of CPPU treatment on fruit appearance **(A)**, color values *a* **(B)** and *b* **(C)** and TSS **(D)**. Shaded horizontal bars indicate commercial harvesting period which started from the end of the 2nd week to the beginning of the 4th week after CPPU treatment. Vertical bars at data points indicate standard error (*n* = 5). Statistical analysis was conducted using Student's *t*-test. *Indicates significant difference between CPPU treatment and the control at *p* < 0.05.

As fruit ripened on tree, the color of the “Feizixiao” fruit pericarp changed from partially red into fully red (Figure 1A). However, the optimal commercial harvest period was not at the fully red ripe stage, obtained 4 WACA, but around 3 WACA (early June), when fruit was still greenish red. The actual harvesting period in the orchard of the experiment initiated at the end of the 2nd week and ended in the 4th week (June 2–12) after CPPU treatment. After the commercial harvesting period, the fully red overripe fruit gradually became purplish red and darkened. As reported previously (Stern et al., 2006; Fahima et al., 2019), fruit coloration was strongly suppressed by CPPU treatment (Figure 1A). Color parameter *a* in the control fruit increased constantly with fruit ripening, turning from a negative value to a positive value between 2 and 3 WACA. It maintained significantly lower in CPPU treatment, where positive value was not attained until 6 WACA (Figure 1B). Color parameter *b* decreased constantly with fruit maturation (Figure 1C) and was initially significantly higher in the control at 1 WACA but became constantly and significantly higher in CPPU treatment in the later sampling dates. Hence, CPPU strongly suppressed the decrease in *b* value. As for TSS, overall, there was no significant difference between CPPU treatment and the control (Figure 1D). TSS remained stable in both CPPU-treated and control fruit for the first 3 WACA but decreased constantly thereafter. The later-on decline was relatively slower in the CPPU treatment. The results agree with the previous reports (Stern et al., 2006; Fahima et al., 2019).

**Figure 2:**
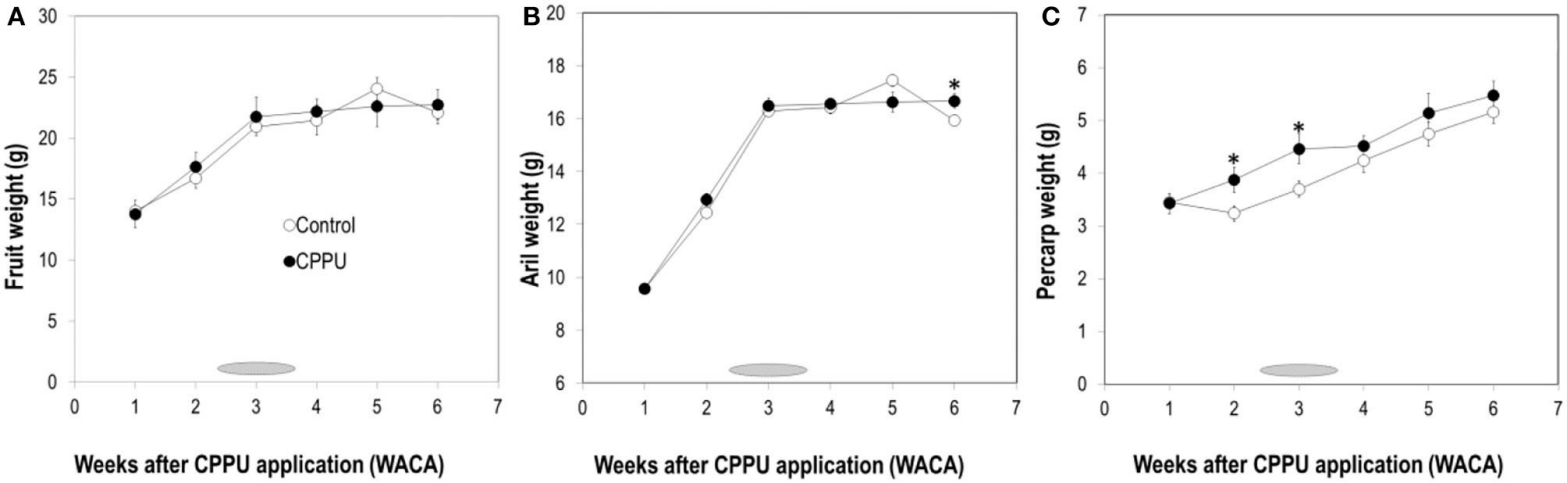
Effect of CPPU treatment on the weights of the fruit **(A)**, aril **(B)**, and pericarp **(C)**. Shaded bar indicates commercial harvesting period; vertical bar at each data point stands for standard error (*n* = 5). *Indicates significant difference between the treatment and the control at *p* < 0.05, Student's *t*-test.

In the control, fruit weight increased with time until 5 WACA, from which fruit weight started to decrease (Figure 2A). CPPU treatment had no significant effect on fruit weight. Aril weight displayed a similar pattern and was not significantly affected by CPPU treatment most of the time (Figure 2B). However, pericarp weight maintained greater in the treatment than in the control throughout the experiment, and the difference was significant at 2 and 3 WACA (Figure 2C). The results showed that 20 mg L^−1^ CPPU sprayed at 7 WAFB is not effective in increasing fruit weight, despite that it increased the pericarp weight.

Based on TSS value and the actual commercial harvest period, the 3rd week after CPPU application was the optimal period for harvesting, and fruit in this period was regarded as being at the full commercial maturity. With this as the starting point or 0 week after full commercial maturity (WAFCM), we traced on-tree changes in quality attributes in overripe fruit.

### Changes in Chlorophylls, Anthocyanins, and Malondialdehyde (MDA) in the Pericarp

**Figure 3:**
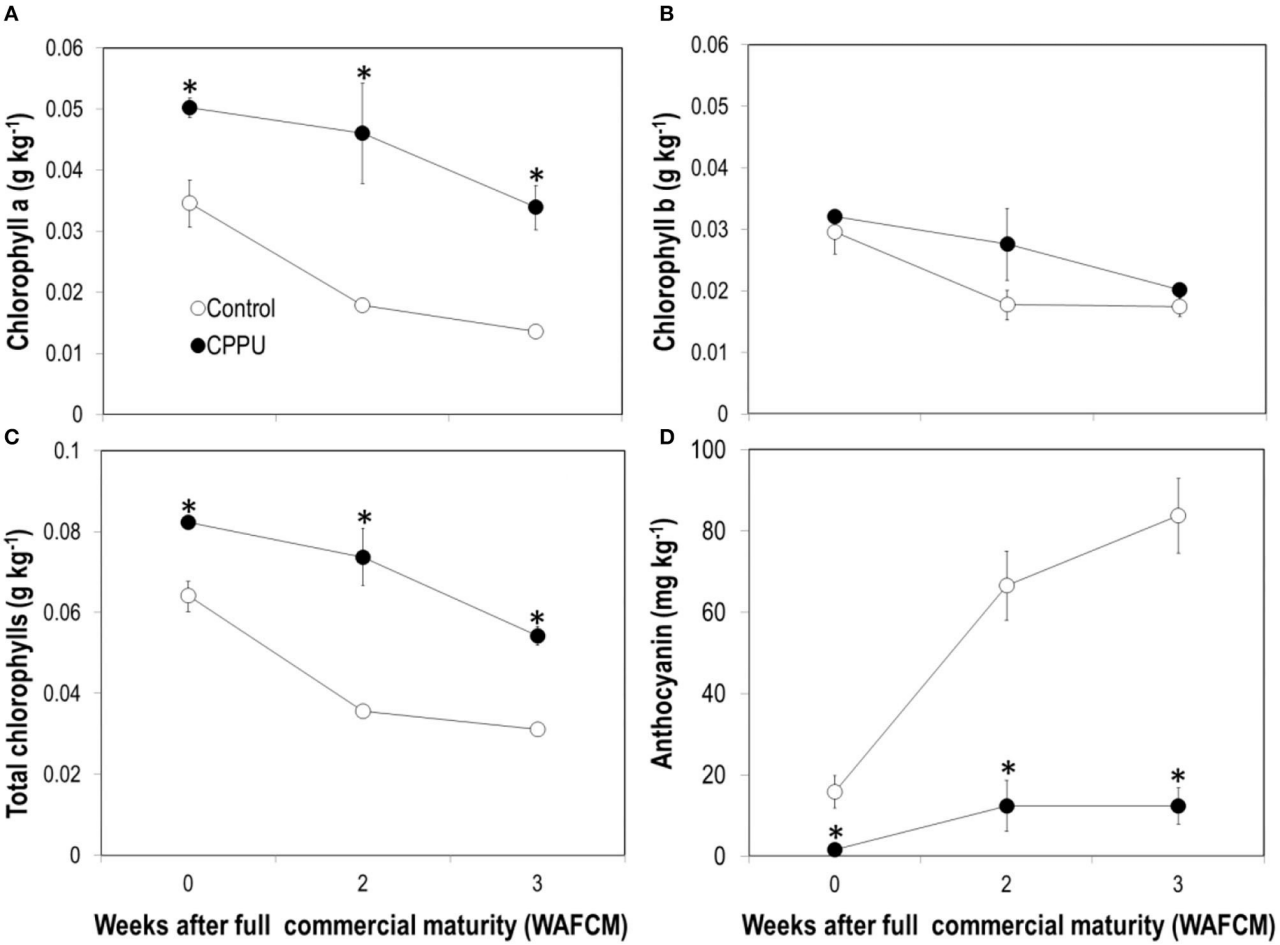
Effect of preharvest application of CPPU on chlorophyll a **(A)**, chlorophyll b **(B)**, total chlorophyll **(C)**, and anthocyanin **(D)** contents in the pericarp of ripe “Feizixiao” litchi fruit remained on tree. Vertical bar at each data point stands for standard error (*n* = 5). *Indicates significant difference between CPPU treatment and the control at *p* < 0.05, Student's *t*-test.

As expected, contents of chlorophyll a, chlorophyll b, and total chlorophylls constantly declined as the fruit became overly ripe (Figures 3A–C). The decline was slower and chlorophyll contents maintained higher in the CPPU treatment. However, there was no significant difference in chlorophyll b whereas significant differences were seen for chlorophyll a, resulting in a significant increase in the ratio of chlorophyll a or chlorophyll b in CPPU treatment fruit pericarp. This result indicates that cytokinin mainly protected chlorophyll a from degradation. In contrast, ripe fruit continued to accumulate anthocyanins as long as they were on tree (Figure 3D), which was in accordance with fruit color change (Figure 1). CPPU strongly suppressed anthocyanin accumulation, which maintained significantly lower in the treatment throughout the experiment. These results suggest that the CPPU treatment caused an unrecoverable inhibition of anthocyanin accumulation.

**Figure 4:**
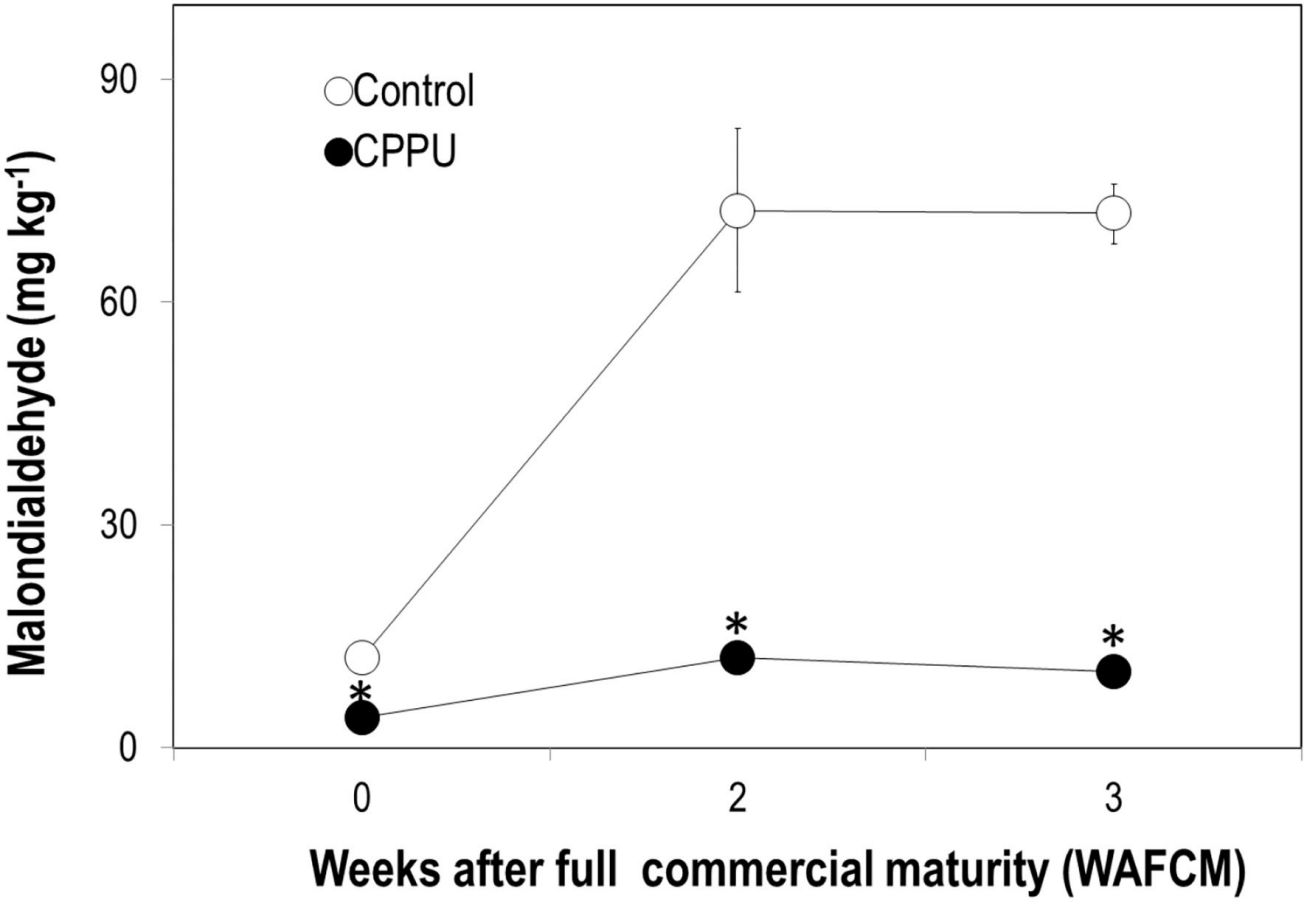
Effect of CPPU treatment on content of malondialdehyde (MDA) in the pericarp. Vertical bar at each data point stands for standard error (*n* = 5). *Indicates significant difference between CPPU treatment and the control at *p* < 0.05, Student's *t*-test.

In the control, there was a sharp accumulation in MDA in the pericarp as the ripe fruit remained on tree within 2 weeks (Figure 4). The MDA content maintained a high level from 2 to 3 WAFCM. In the CPPU treatment, MDA content in the pericarp also increased within 2 WAFCM. However, CPPU strongly reduced its accumulation compared with the control (Figure 4).

### Changes in Non-Anthocyanin Flavonoids in the Pericarp and the Aril

**Figure 5:**
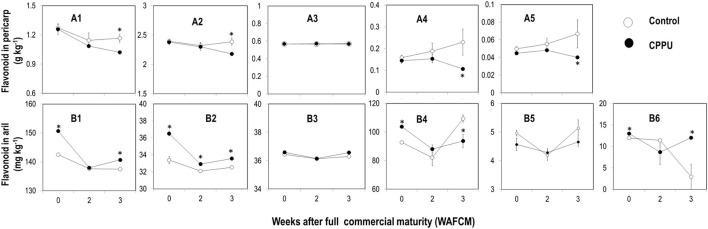
Effect of preharvest application of CPPU on changes in some non-anthocyanin flavonoids including epicatechin **(A1,B1)**, gallocatechin **(A2,B2)**, epicatechin gallate **(A3,B3)**, proanthocyanidin B2 **(A4,B4)**, keampfenol-3-o-rutincide **(A5,B5)**, and proanthocyanidin B1 **(B6)** in the pericarp (A series) and the aril (B series) of ripe “Feizixiao” litchi fruit on tree. Vertical bar at each data point stands for standard error (*n* = 5). *Indicates significant difference between CPPU treatment and the control at *p* < 0.05, Student's *t*-test.

Using HPLC, we were able to detect five non-anthocyanin flavonoids in the pericarp including epicatechin, gallocatechin, epicatechin gallate, proanthocyanidin B2, and keampfenol-3-o-rutincide, and in the aril, one more flavonoid, proanthocyanidin B1 was detected (Figure 5). These non-anthocyanin flavonoids were all higher in the pericarp than in the aril.

In the pericarp, epicatechin and gallocatechin were the most abundant flavonoids, and all the five flavonoids maintained relatively stable in the control (Figures 5A1–A5). At full commercial maturity (0 WAFCM), CPPU treatment showed no significant effect on these flavonoids, but as the fruit became overripe, it tended to decrease epicatechin, gallocatechin, proanthocyanidin B2, and keampfenol-3-o-rutincid, which became significantly lower in CPPU treatment than in the control at 3 WAFCM.

In the aril, epicatechin and proanthocyanidin B2 were the dominating flavonoids. Except for proanthocyanidin B1 (Figure 5B6), which decreased as the fruit became overripe, all the other flavonoids maintained relatively stable. CPPU significantly increased the levels of epicatechin, proanthocyanidins, and gallocatechin in the aril at 0 WAFCM. However, the treatment had no significant effect on epicatechin gallate and keampfenol-3-o-rutincide. The results suggest that the CPPU treatment increased the accumulation of major tannin components in the aril.

### Changes in Sugars, Anaerobic Respiration Products, and Organic Acids in the Aril

**Figure 6:**
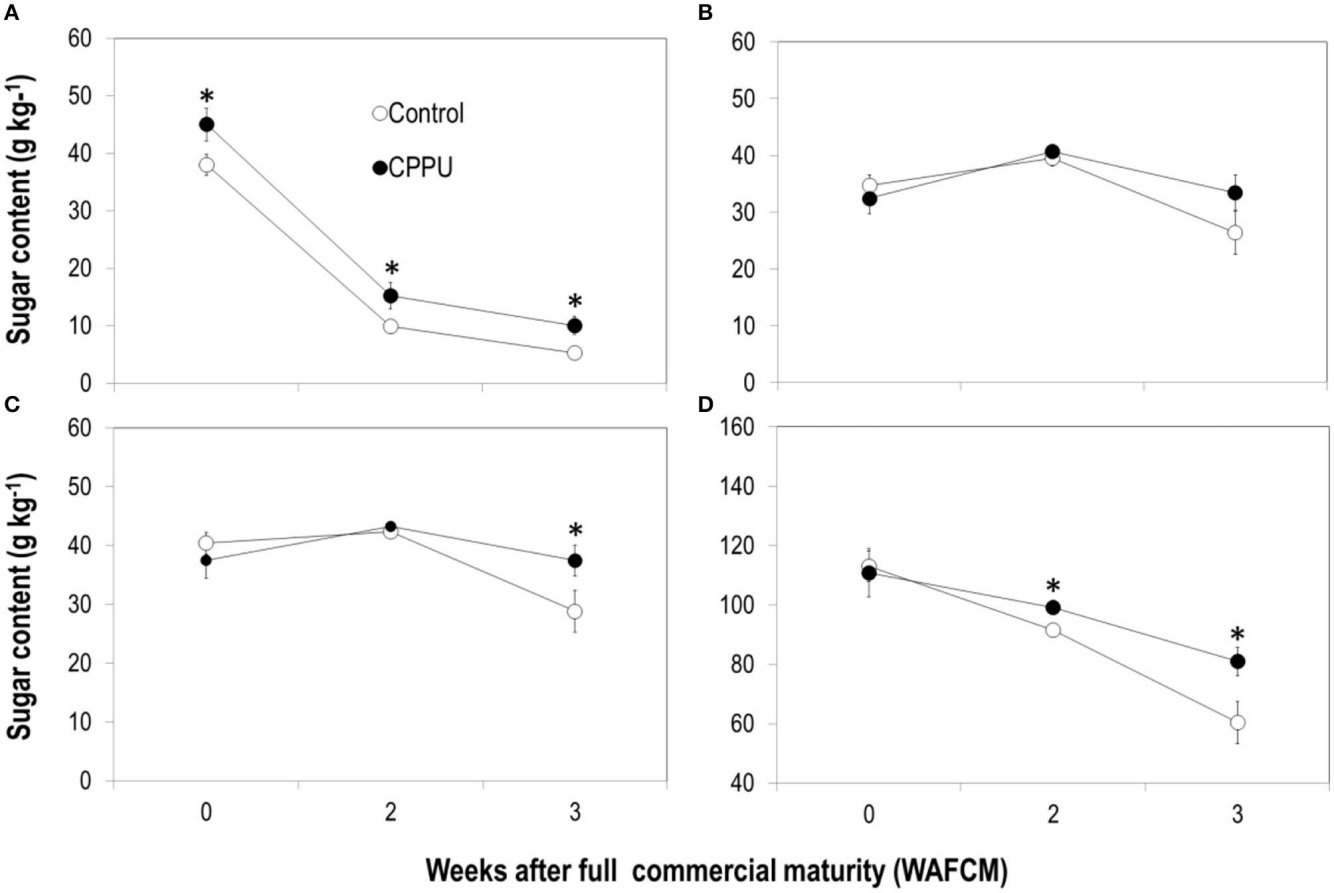
Effect of preharvest application of CPPU on changes in sucrose **(A)**, glucose **(B)**, fructose **(C)**, and total sugars **(D)** in the aril of the ripe “Feizixiao” litchi fruit remained on tree. Vertical bar at each data point stands for standard error (*n* = 5). *Indicates significant difference between CPPU treatment and the control at *p* < 0.05, Student's *t*-test.

In both the CPPU treatment and the control fruits, sucrose in the aril decreased constantly as the fruit became overripe. Yet, reducing sugars, that is, fructose and glucose, displayed a slight increase until 2 WAFCM and then decreased (Figures 6A–C). The CPPU treatment increased sucrose content and slowed its decline and slightly increased reducing sugars at 3 WAFCM. Total sugar, the sum of sucrose and reducing sugars, declined constantly as the fruit became overripe (Figure 6D), in accordance with the pattern detected for TSS (Figure 1). There was no significant difference in total sugar between the control and the CPPU treatment at 0 WAFCM, but the CPPU treatment slowed sugar decline and thus had a significantly higher total sugar content in overripe fruit compared with the control (Figure 6D).

**Figure 7:**
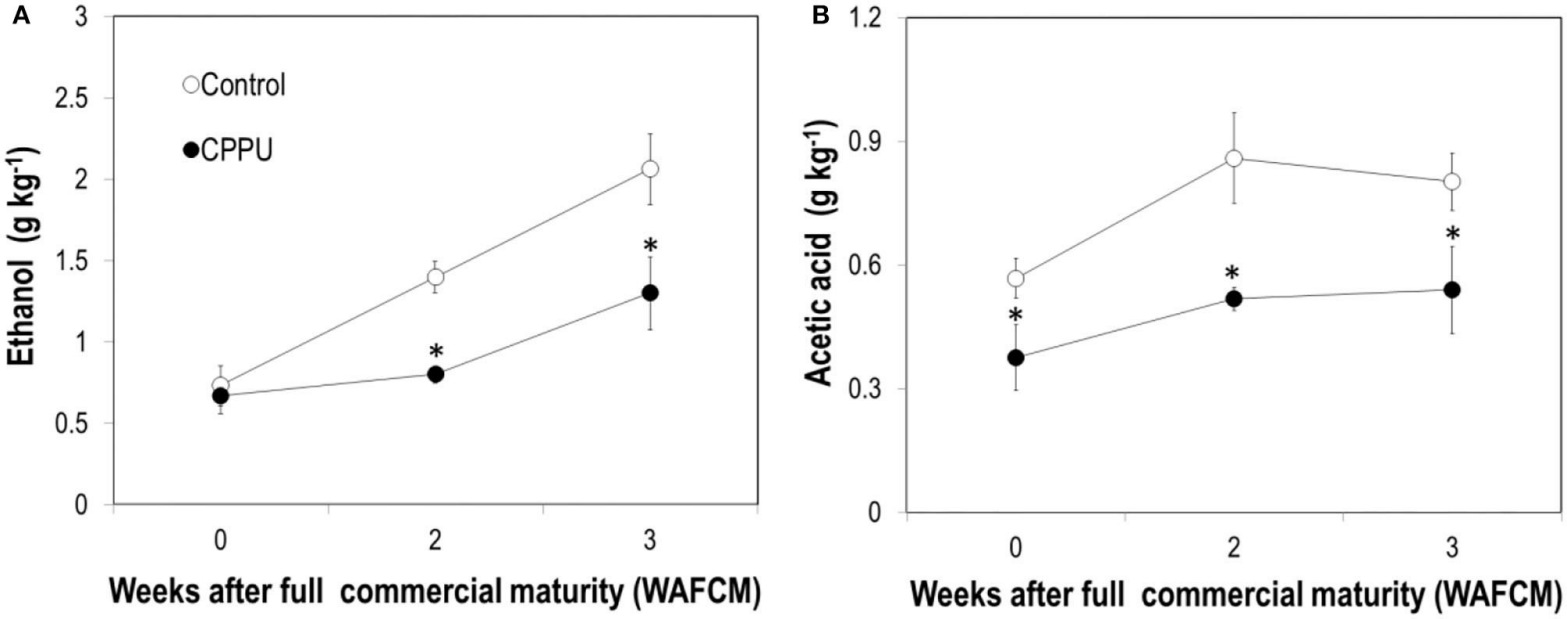
Effect of preharvest application of CPPU on changes in ethanol **(A)** and acetic acid **(B)** in the aril of the ripe “Feizixiao” litchi fruit remained on tree. Vertical bar at each data point stands for standard error (*n* = 5). *Indicates significant difference between CPPU treatment and the control at *p* < 0.05, Student's *t*-test.

The aril of ripe fruit constantly accumulated ethanol as they hanged on the tree, and CPPU treatment significantly suppressed ethanol accumulation in the aril compared with the control (Figure 7A). Acetic acid also increased in overripe control fruit and was significantly lower in the CPPU treatment (Figure 7B). The results suggest that the CPPU treatment was effective in suppressing anaerobic respiration.

**Figure 8:**
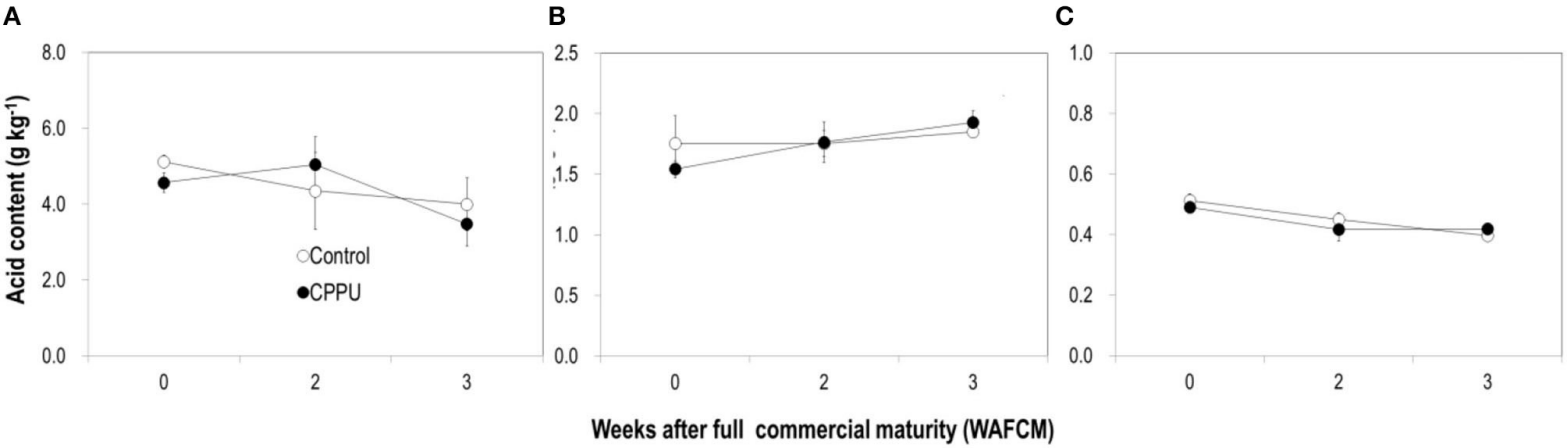
Effect of preharvest application of CPPU on changes in organic acids including malic acid **(A)**, tartaric acid **(B)** and ascorbic acid **(C)** in the aril of the ripe “Feizixiao” litchi fruit remained on tree. Vertical bar at each data point stands for standard error.

In addition, we also analyzed the contents and composition of organic acids in the aril. The dominating organic acids in litchi fruit aril tissue are malic acid, tartaric acid, and ascorbic acid (Wang et al., 2006). Malic acid and ascorbic acid declined as the fruit became overripe, whereas tartaric acid maintained relatively stable (Figure 8). The CPPU treatment had no significant effect on the content of any of these organic acids (Figure 8).

### Expression of Invertase Genes and Enzyme Activity in the Aril

**Figure 9:**
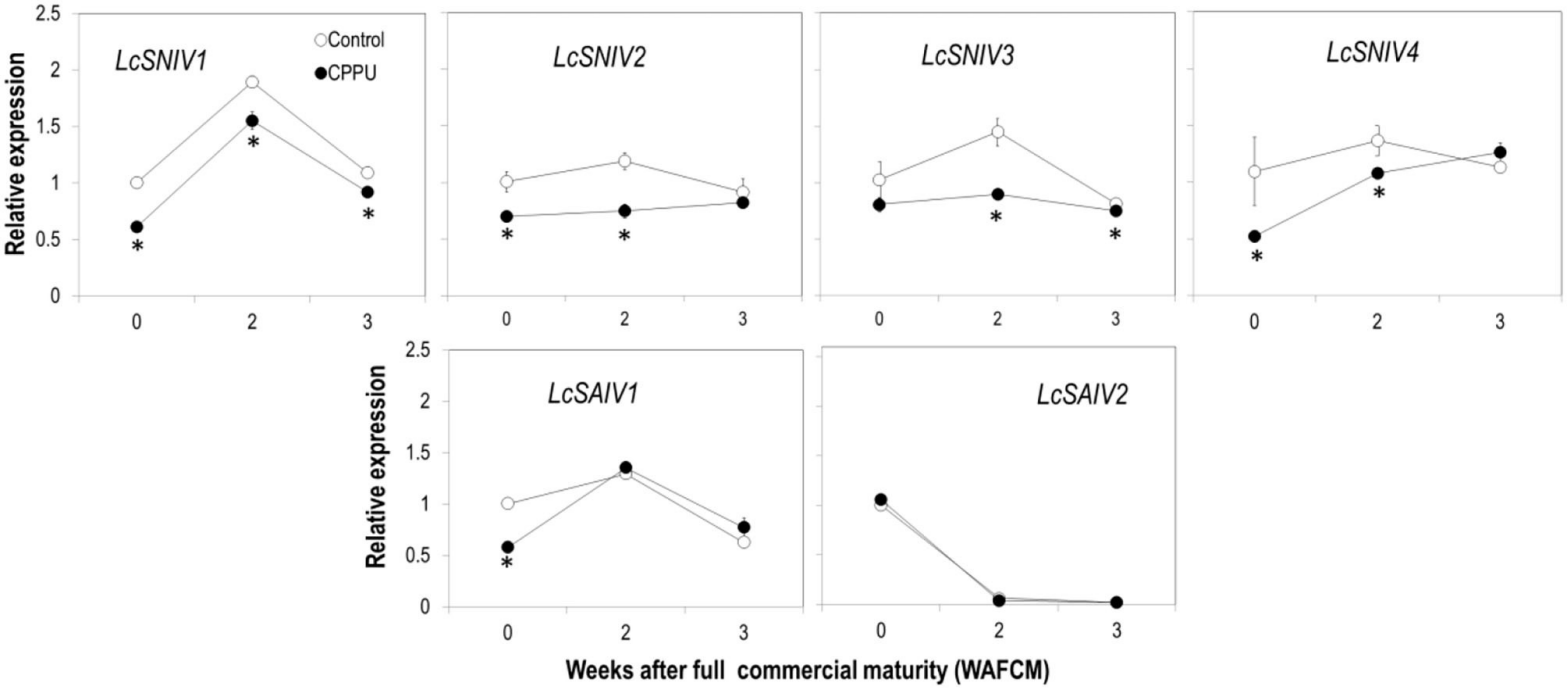
Effects of preharvest application of CPPU on expression of genes encoding SNIVs (*LcSNIV1, LcSNIV2, LcSNIV3*, and *LcSNIV4*) and SAIVs (*LcSAIV1* and *LcSAIV2*). Vertical bar at each data point stands for standard error (*n* = 5). *Indicates significant difference between CPPU treatment and the control at *p* < 0.05, Student's *t*-test.

The effect of CPPU on the expression of the key genes involved in sucrose hydrolysis in the aril tissue was examined. The overall expression pattern of the four SNIV genes (*LcSNIV1, LcSNIV2, LcSNIV3*, and *LcSNIV4*) was similar, and their expression was significantly inhibited by CPPU treatment (Figure 9). The expression patterns of the two SAIV genes (*LcSAIV1* and *LcSAIV2*) were quite different. The highest *LcSAIV1* expression was detected at 2 WAFCM, whereas *LcSAIV2* expression was highest at 0 WAFCM and decreased dramatically as the fruit became overripe. CPPU treatment significantly reduced the gene activity of *LcSAIV1* at 0 WAFCM but had no significant effect on later stages. The treatment significantly reduced *LcSAIV2* activity at 2 WAFCM, but showed no effect at other stages.

**Figure 10:**
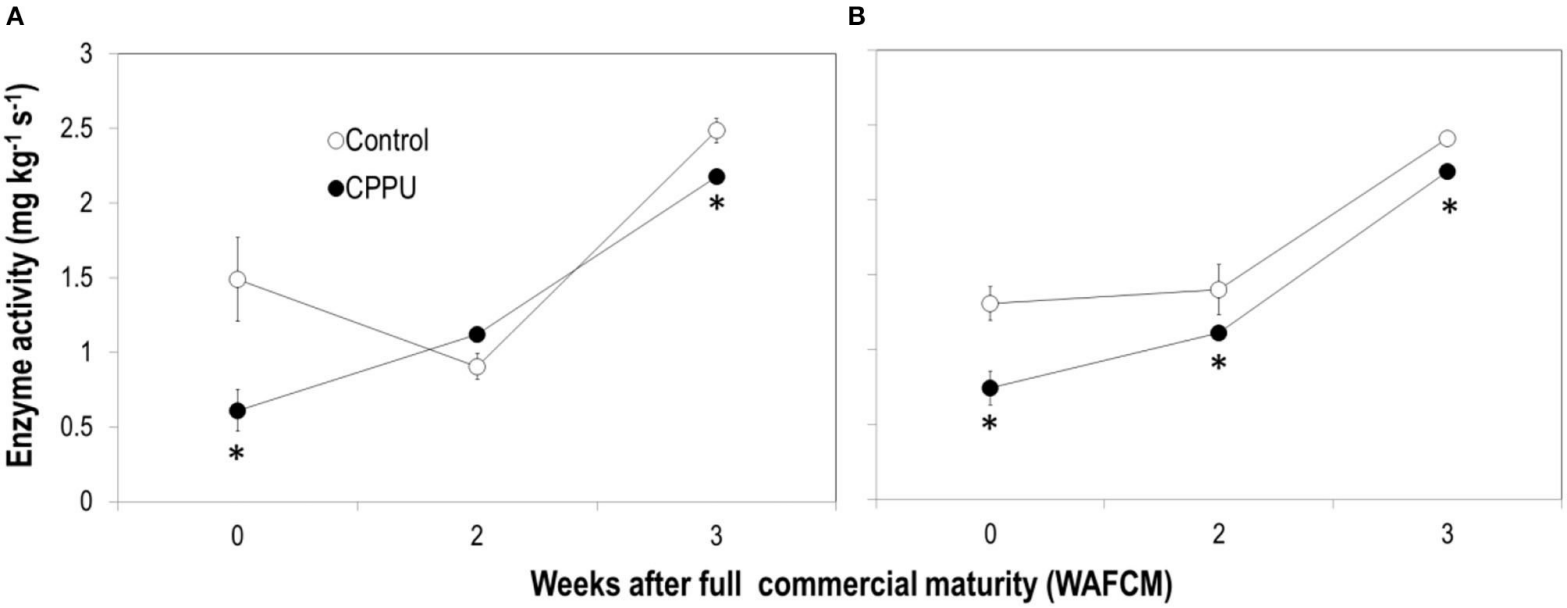
Effects of preharvest application of CPPU on the activities of SNIV **(A)** and SAIV **(B)**. Vertical bar at each data point stands for standard error (*n* = 5). *Indicates significant difference between CPPU treatment and the control at *p* < 0.05, Student's *t*-test.

The enzymatic activities of both SNIV and SAIV tended to increase as the fruit became overripe on the tree (Figure 10), differing from the change pattern of their gene expression. CPPU treatment significantly lowered the activity of SNIV at 0 and 3 WAFCM and that of SAIV throughout the experiment.

### Expression of PDC and ADH Genes and Enzyme Activity in the Aril

**Figure 11:**
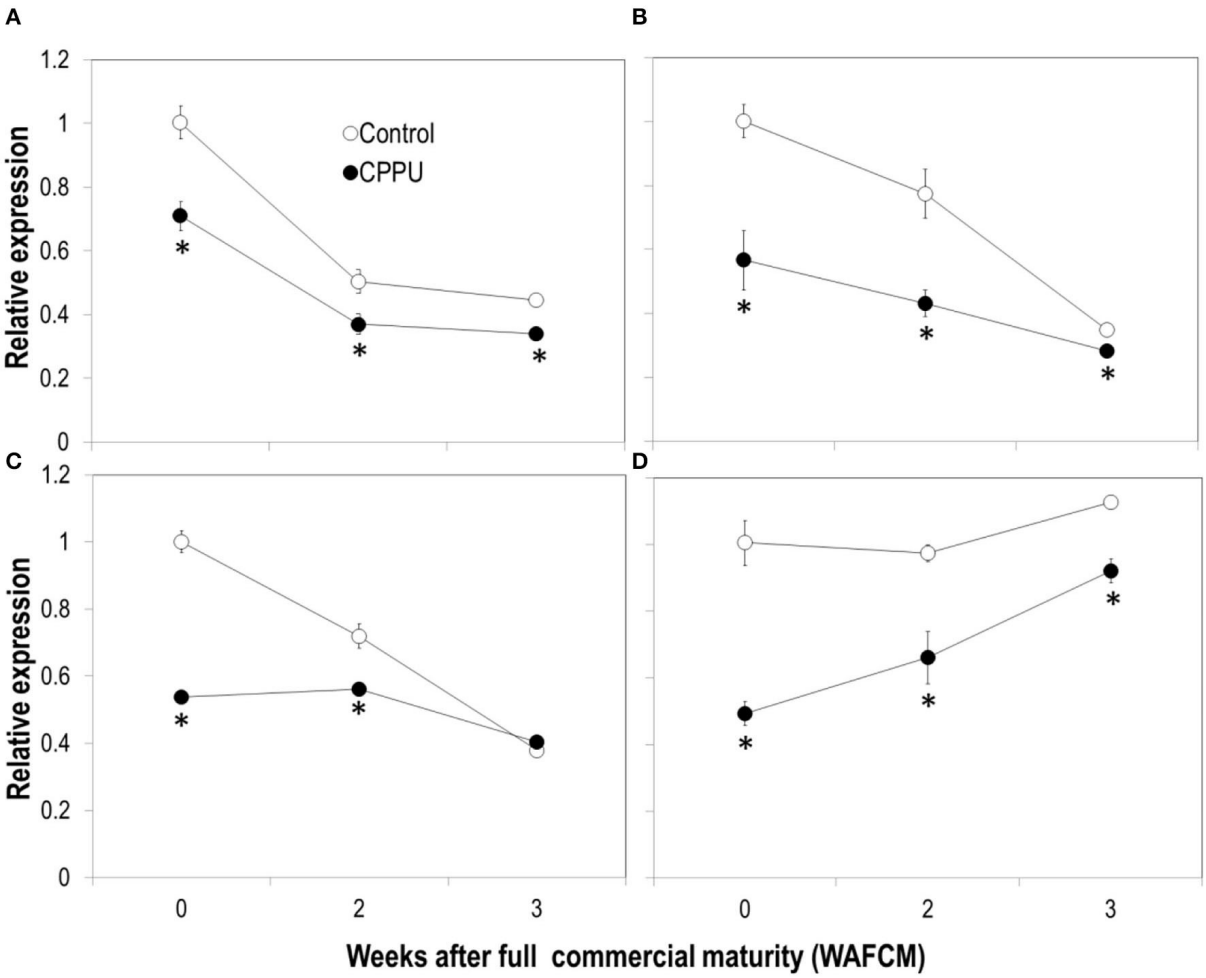
Effects of preharvest application of CPPU on expression of genes encoding pyruvic decarboxylase (PDC) [**(A)** *LcPDC1*; **(B)** *LcPDC2*] and ADH [**(C)** *LcADH1*, **(D)** *LcADHL1*]. Vertical bar at each data point stands for standard error (*n* = 5). *Indicates significant difference between CPPU treatment and the control at *p* < 0.05, Student's *t*-test.

Expression of the two genes encoding LcPDC1 (LITCHI029074) and LcPDC2 (LITCHI018164) and two genes encoding ADH (LITCHI007374, *LcADH1*) or ADH-like (LITCHI003007, *LcADHL1*) was analyzed through qPCR (Figure 11). The pattern of the two PDC genes was similar, decreasing as the fruit became overripe in both the treatment and the control. CPPU significantly suppressed the expression of these two genes. The expression of *LcADH1* decreased constantly, whereas *LcADHL1* expression maintained relatively constant as the fruit became overripe. The expression level of these two genes was significantly lower in CPPU-treated fruit throughout the experiment.

**Figure 12:**
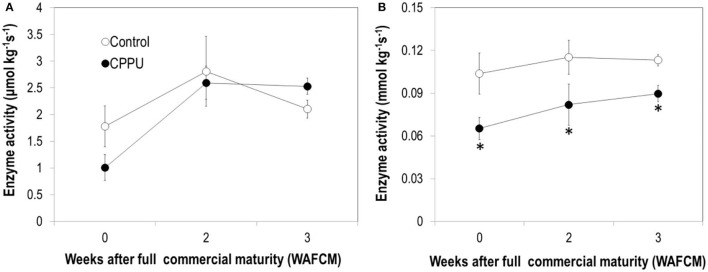
Effects of preharvest application of CPPU on the activities of pyruvic decarboxylase (PDC) **(A)** and alcohol dehydrogenase (ADH) **(B)**. Vertical bar at each data point stands for standard error (*n* = 5). *Indicates significant difference between CPPU treatment and the control at *p* < 0.05, Student's *t*-test.

Pyruvic decarboxylase activity maintained relatively stable in the aril of the control fruit, which is different from the constantly decreasing pattern of the corresponding gene expression. CPPU slightly reduced PDC activity at 0 WAFCM, but had no significant difference from the control throughout the experiment (Figure 12A). The activity of ADH maintained relatively stable and was significantly lowered by the CPPU treatment (Figure 12B). The effect of CPPU on ADH activity agrees with its effect at the level of *ADH* gene expression.

These results suggest that CPPU downregulated the pathway of anaerobic respiration in the aril of ripe litchi.

## Discussion

### Dynamics of CPPU Residue in the Pericarp and the Aril

As a plant growth regulator with strong cytokinin activity, CPPU has been extensively used to promote cell division and thus increase fruit size. Because of the very low concentrations of CPPU applied, residue of CPPU in the fruit flesh is generally below the maximum residue limit (MRL; Chen et al., 2013; Li et al., 2020). However, the potential health risk of CPPU is still under evaluation (Bu et al., 2019). Available reports have shown that the dissipation rate of CPPU residue in fruit differs greatly among species (Zhang et al., 2011; Chen et al., 2013; Li et al., 2020). The half-life of CPPU residue was 15.8–23.0 days in citrus fruits (Chen et al., 2013), 11.9 days in kiwi fruit (Li et al., 2020), and 1.5 days in muskmelon (Zhang et al., 2011). In this study, after application of CPPU at 20 mg L^−1^, residue in litchi fruit reduced by 76.3% in the first 2 weeks, whereas the second 2 weeks saw only 26.7% reduction in CPPU residue level (Table 1). These results suggest that the half-life of CPPU residue in litchi fruit is shorter than 14 days and that dissipation rate declines with time. CPPU residue in the pericarp was far higher than in the aril tissue. The concentration decreased with time in both tissues. Interestingly, the per-fruit amount of CPPU residue in the pericarp and the aril displayed opposite trends, constantly decreasing in the pericarp but constantly increasing in the aril. These results suggest that diffusion of CPPU from the pericarp to the aril occurred constantly and that degradation of CPPU residue might have taken place only in the pericarp but not in the aril. This in turn may suggest that the degradation of CPPU might be dependent on light and/or air exposure. Throughout the experiment, the concentration of CPPU residues in the aril, the edible part, was lower than 10 μg kg^−1^, MRL in Australia and the USA, and far lower than the MRL in Japan (100 μg kg^−1^; USDA database for Maximun Residue Limits, http://www.mrldatabase.com). Therefore, in terms of MRL, it is acceptable to apply CPPU at 20 mg L^−1^ to litchi fruit.

### CPPU Suppresses Certain Events of the Ripening Process in Litchi

Cytokinins are generally considered as a suppressor of fruit ripening (NeSmith, 2002; Wang et al., 2007; Peppi and Fidelibus, 2008). However, studies have shown that exogenous cytokinin, CPPU, has differential effect on fruit ripening among species. CPPU suppresses multiple events in ripening process of grape (Peppi and Fidelibus, 2008; Maoz et al., 2014) and blueberry (NeSmith, 2002) including coloration, softening, soluble solid accumulation, and tannin loss, whereas in kiwifruit, CPPU advances such ripening events as fruit softening, sugar accumulation, and flesh coloration (Lötter, 1992). In apple, CPPU application increased fruit size with no significant effect in soluble solid content (Kazuhiro et al., 2018). In litchi, CPPU is effective to delay coloration (Stern et al., 2006; Fahima et al., 2019; Figure 1), with downregulation of anthocyanin biosynthesis (*LcMyB1* and *LcUFGT*) and chlorophyll degradation genes (*LcSGR*) and upregulation of chlorophyll biosynthesis genes (Wei et al., 2011; Lai et al., 2015). However, CPPU treatment did not significantly affect soluble solutes, acidity, and fruit weight (Fahima et al., 2019). This study further shows differential effect of CPPU on ripening events. While significantly suppressing the decline in chlorophyll levels and anthocyanin accumulation in the pericarp, CPPU showed no significant effect on contents of total soluble sugars in the aril at full commercial maturity. Similar effects of dipping treatment with another cytokinin, 6-benzyl aminopurine (6-BA) at 100 mg L^−1^, on litchi ripening events were reported in a previous study (Wang et al., 2007). Since neither aril size nor total sugar content at full commercial maturity was significantly affected by CPPU treatment, sugar import to the aril seems not a target of cytokinin regulation in litchi. However, CPPU treatment induced sugar compositional change with a significant increase in sucrose (Figure 6), indicating that sucrose conversion is under cytokinin regulation, which will be discussed later.

CPPU had no significant effect on fruit acidity in litchi (Fahima et al., 2019), grape (Peppi and Fidelibus, 2008), highbush blueberry (Fujisawa et al., 2018), and apricots (Roussos et al., 2021). This study further shows that neither quantity and nor composition of major organic acids in litchi aril was affected by CPPU treatment (Figure 8). Hence, acid metabolism during fruit ripening seems beyond the targets of cytokinin regulation in litchi.

Non-anthocyanin flavonoids such as epicatechins and proanthocyanidins (condensed tannins) are the major contributors to astringency taste. These are the major phenolic components in litchi pericarp (Sarni-Manchado et al., 2000) and aril (Zhang et al., 2013). CPPU-treated grape berries maintained higher levels of condensed tannins and thus were more astringent than untreated berries (Maoz et al., 2014). In our study, we found differential effect of CPPU on these flavonoids between the pericarp and the aril of litchi. In the pericarp of mature litchi fruit, CPPU had no significant effect on all tested non-anthocyanin flavonoids including epicatechin, gallocatechin, epicatechin gallate, proanthocyanidin B1, and keampfenol-3-o-rutincide (Figure 5). However, in the aril tissue, CPPU treatment significantly increased some non-anthocyanin flavonoids such as epicatechin, gallocatechin, and proanthocyanidins B1 and B2 at full commercial maturity (Figure 5), which is similar to the effect observed in grapes (Maoz et al., 2014). These results indicate that flavonoid metabolism in the pericarp and the aril might be subjected to differential regulation mechanism, which awaits further exploration.

Within the pericarp, CPPU strongly suppressed anthocyanin accumulation while hardly affected non-anthocyanin flavonoids. The biosynthesis of anthocyanins and non-anthocyanin flavonoids occurs through a largely shared pathway. The “turning point” toward anthocyanin biosynthesis occurs at the conversion from anthocyanidins to anthocyanins catalyzed by UDPGLUCOSE: FLAVANOID GLUCOSYLTRANSFERASE (UFGT; Wang et al., 2004). It was previously shown that CPPU strongly inhibited the expression of *LcUFGT*, whereas it had no significant effect on the genes involved in the upstream part of the flavonoid biosynthetic pathway, such as *LcCHS, LcCHI, LcF3H, LcDFR*, and *LcANS* (Wei et al., 2011). This explains why CPPU strongly suppresses anthocyanin accumulation without affecting non-anthocyanin flavonoid levels in the pericarp.

From the above discussion, it can be concluded that cytokinin partially suppresses the ripening process in litchi.

### CPPU Improves On-Tree Quality Maintenance in Ripe Litchi Fruit

The senescence-delaying effect of cytokinin is well-known. Treatment of cytokinin delays leaf senescence *via* downregulating senescence-associated genes (SAGs) (Zwack and Rashotte, 2013) and inhibits expression of genes involved in transformation of chloroplast to chromoplast (Robson et al., 2004; Zubo et al., 2008). Fruit ripening can be regarded as the early phase of fruit senescence involving degradation of chloroplast similar to leaf senescence (Huang and Wu, 2006). The follow-up senescence process of ripe litchi is very rapid even on tree, with loss of sugars (Wang et al., 2003), development of off-flavor due to the accumulation of alcohol and acetaldehyde (Pesis et al., 2002), loss of disease resistance (Huang and Wu, 2006), and worsened storability (Huang et al., 2005), giving litchi a very short “hanging life” and harvest period. Similar to the suppressing effect on leaf senescence (Robson et al., 2004), cytokinin is effective to delay senescence of fruits. CPPU application during fruit development has been reported to enhance disease resistance and postharvest performance in grape (Ben-Arie et al., 1997), pitaya (Jiang et al., 2020), highbush blueberry (Jorge et al., 2014), and litchi (Stern et al., 2006). CPPU-treated litchi fruit maintained growth and had a slower coloration process and extended hanging life and also better postharvest performance (Stern et al., 2006). This study examined the effect of preharvest CPPU treatment on on-tree changes in important components related to fruit quality in the aril of mature litchi fruit and obtained some novel findings.

First, overripe litchi accumulated significant amounts of MDA in the pericarp (Figure 4), an indicator of lipid peroxidation. CPPU strongly suppressed MDA accumulation, indicating that cytokinin inhibits lipid peroxidation and thus may help maintaining membrane integrity in litchi.

Second, CPPU significantly suppressed sugar decline in overripe fruit. The treatment resulted in a constantly higher sucrose level compared to the control, whereas reducing sugar (fructose and glucose) levels were not significantly affected. The higher total sugars in CPPU treatment were thus largely contributed by higher sucrose levels maintained. This result indicates that CPPU might have suppressed the conversion of sucrose to reducing sugars, which is catalyzed by neutral or acid invertases. A previous study showed that CPPU promoted the activities of acid and neutral invertases when applied at fruit set but had no significant effect in later stages in muskmelon (Hayata et al., 2001). In contrast, our result showed that CPPU significantly decreased the activities of both SNIV and SAIV in the aril of ripe litchi fruit (Figure 10), which explains the higher sucrose level detected in CPPU-treated fruit. In line with the reduced SNIV activity, CPPU significantly reduced the expression of all the neutral invertase genes tested (*LcSNIV1, LcSNIV3*, and *LcSNIV4*), indicating that their expression is subject to transcriptional regulation by cytokinin. Although CPPU significantly reduced the expression of two acid invertase genes (*LcSAIV1* and *LcSAINV2*) only at certain time points, it constantly inhibited the enzymatic activity. This result indicates that CPPU may inhibit acid invertase transcriptionally and non-transcriptionally.

Third, similar to the results obtained by Pesis et al. (2002), the aril of litchi accumulated anaerobic products such as alcohols and acetic acid as the fruit became overripe. Accumulation of alcohol was also reported in ripe mandarins (Hijaz et al., 2020). The development of watercore associated with fruit ripening in pear was found to be related to the occurrence of anaerobic respiration (Liu et al., 2021). Our study provided the first-hand evidence that CPPU significantly inhibits the accumulation of alcohol and acetic acid in overripe fruit, indicating that cytokinin prevents the occurrence of anaerobic respiration in litchi aril. The activity of ADH, one of the key enzymes in anaerobic respiration, was significantly reduced by CPPU treatment, and likewise, the gene expression was downregulated by the treatment. Activity of PDC, another key enzyme in anaerobic respiration, increased as the fruit became overripe, which was opposite to the decreasing expression of the corresponding genes, indicating that PDC activity in litchi aril is not entirely determined at the level of gene transcription. CPPU slightly reduced PDC activity in ripe litchi and significantly reduced the transcript levels of the two PDC genes. These results suggest that cytokinin suppresses anaerobic respiration *via* transcriptional regulation.

Studies have shown that astringency disappearance during fruit ripening is largely due to coagulation of soluble tannins leading to loss of their solubility, which is mediated by an anaerobic respiration product, acetaldehyde (Tamura et al., 1999), and can be induced by hypoxia condition (Jamil et al., 2019) and alcohol treatment (Tamura et al., 1999). The loss of astringency in litchi aril is likely associated with the occurrence of anaerobic respiration during fruit ripening, reflected by constant accumulation of ethanol and acetaldehyde (Pesis et al., 2002). CPPU suppressing anaerobic respiration in litchi aril provides a reasonable explanation to the finding that condensed tannins in the aril of the treated fruit remained higher compared to control fruit.

## Conclusions

Residue of CPPU sprayed at 20 mg L^−1^ on fruit surface dissipated fast, and over 76% was lost in the first 2 weeks. The residue concentration in the flesh tissue maintained lower than 10 ug kg^−1^ and below the MRL in many countries. Cytokinin inhibits certain aspects of ripening process in litchi, suppressing chlorophyll degradation and anthocyanin accumulation in the pericarp, but showing no significant effect on sugar accumulation and acid metabolism in the aril during fruit ripening. However, cytokinin is effective to delay the senescence of ripe litchi fruit *via* suppressing lipid peroxidation in the pericarp and slow sugar decline *via* inhibiting sucrose hydrolysis and anaerobic respiration transcriptionally and/or non-transcriptionally in the aril. Therefore, cytokinin treatment helps to maintain quality of ripe litchi fruit and improves the performance of “on-tree storage” with an extended hanging life.

**Figure 13:**
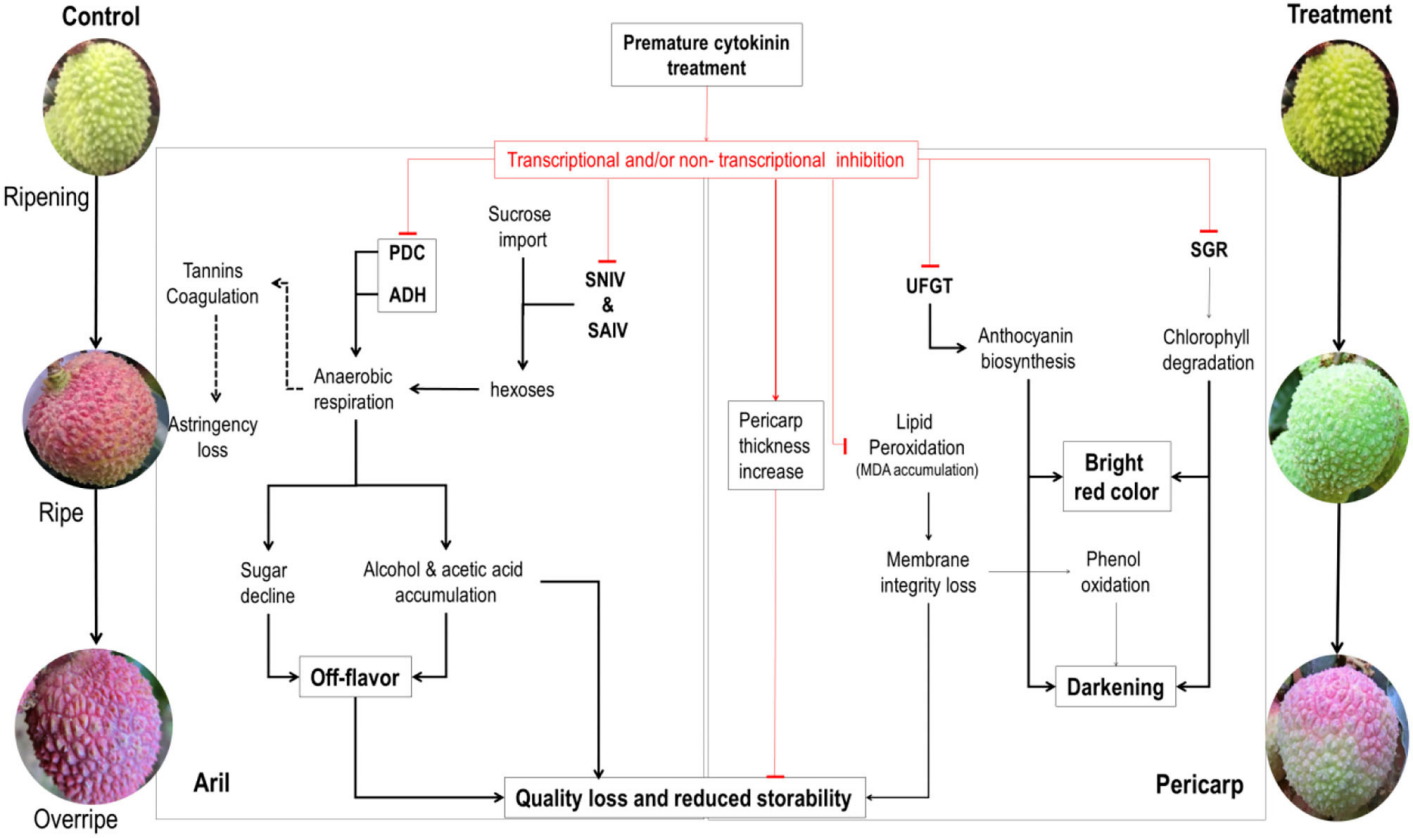
Schematic mode of the effect of cytokinin on the physiochemical changes in the pericarp and the aril during maturation and senescence of litchi fruit on tree. The black lines with an arrow indicate the natural processes involved in fruit ripening and senescence. Dashed lines indicate effects to be proved. Red lines ended with a top bar indicate the inhibitory effect exerted by CPPU, whereas arrowed red line indicates promoting effect of CPPU. PDC, pyruvate decarboxylase; ADH, alcohol dehydrogenase; UFGT, flavonoid glucosyltransferase; UDPG, uridine diphosphoglucose; SGR, STAY-GREEN; SNIV, soluble neutral invertase; SAIV, soluble acid invertase.

A schematic mode showing the physiochemical changes in the pericarp and the aril during fruit maturation and senescence of litchi and how cytokinin regulates them is shown in Figure 13.

## Data Availability Statement

The original contributions presented in the study are included in the article/Supplementary Material, further inquiries can be directed to the corresponding author/s.

## Author Contributions

X-SL: conducted investigation, data curation, validation, and writing original draft. Y-CL: involved in investigation and data curation. S-WW: carried out investigation, methodology, and data curation. H-CW: involved in conceptualization, methodology, and funding acquisition. SH-S: performed conceptualization, writing, reviewing, and editing. X-MH: carried out conceptualization, supervision, visualization, writing, reviewing, editing, project administration, funding acquisition, and provided resources. All authors contributed to the article and approved the submitted version.

## Funding

This study has been supported by National Natural Science Foundation of China (31772248), the National Key Research and Development Program (2018YFD1000200), and the China Litchi and Longan Industry Technology Research System (Project No. CARS-32-11).

## Conflict of Interest

The authors declare that the research was conducted in the absence of any commercial or financial relationships that could be construed as a potential conflict of interest.

## Publisher's Note

All claims expressed in this article are solely those of the authors and do not necessarily represent those of their affiliated organizations, or those of the publisher, the editors and the reviewers. Any product that may be evaluated in this article, or claim that may be made by its manufacturer, is not guaranteed or endorsed by the publisher.

## Supplementary Material

Supplementary Figure 1 Effect of preharvest CPPU application at different concentrations on TSS content in fruit at commercial maturity and 2 weeks later. Vertical bars on the top of columns indicate standard error (*n* = 5). ^*^Indicates significant difference among treatments at the same sampling time at *p* < 0.05, Duncan's multiple range test. Different letters behind the TSS reduction values indicate significant difference among treatments at *p* < 0.05, Duncan's multiple range test.

Supplementary Table 1 Primer sequences of selected genes for qPCR analysis.
