# Supplementary figures and images for: Residue Analysis and the Effect of Preharvest Forchlorfenuron (CPPU) Application on On-Tree Quality Maintenance of Ripe Fruit in “Feizixiao” Litchi (Litchi chinensis Sonn.)
Source: Front Plant Sci. 2022 Mar 4;13:829635. doi: 10.3389/fpls.2022.829635 (PMC8931753; doi:10.3389/fpls.2022.829635)

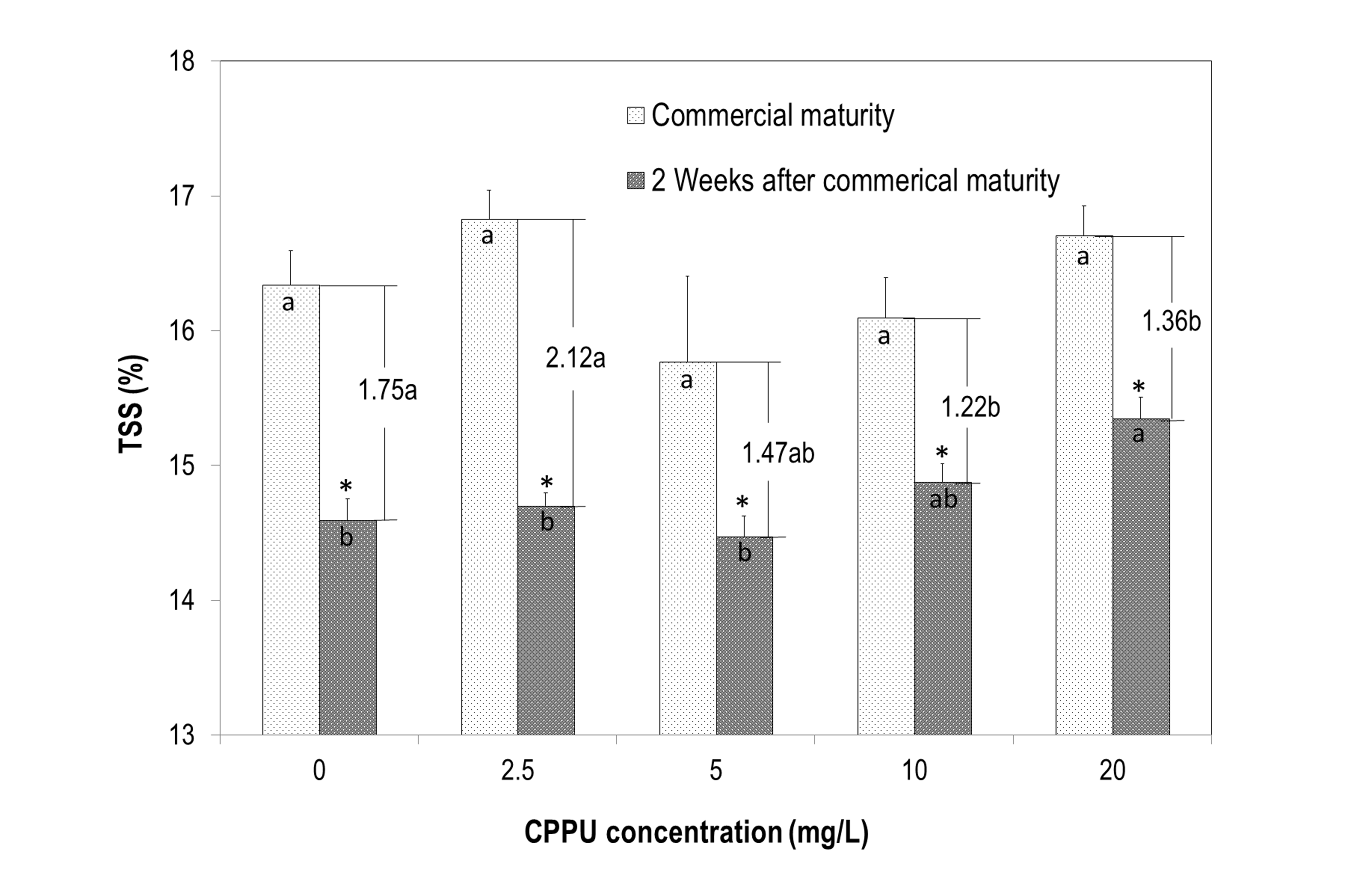

Supplement: Supplementary Figure 1 — Effect of preharvest CPPU application at different concentrations on TSS content in fruit at commercial maturity and 2 weeks later. Vertical bars on the top of columns indicate standard error (n = 5). *Indicates significant difference among treatments at the same sampling time at p < 0.05, Duncan's multiple range test. Different letters behind the TSS reduction values indicate significant difference among treatments at p < 0.05, Duncan's multiple range test. [file Image_1.TIF]
