# Supplementary material for: Residue Analysis and the Effect of Preharvest Forchlorfenuron (CPPU) Application on On-Tree Quality Maintenance of Ripe Fruit in “Feizixiao” Litchi (Litchi chinensis Sonn.)
Source: Front Plant Sci. 2022 Mar 4;13:829635. doi: 10.3389/fpls.2022.829635 (PMC8931753; doi:10.3389/fpls.2022.829635)
Supplement: Supplementary Table 1 — Primer sequences of selected genes for qPCR analysis. [file Table_1.DOCX]

**Additional files**

**Table S1** Primer sequences of selected genes for Q-PCR analysis

| Code | id in genome | Description | Forward primer | Downstream primers |
| --- | --- | --- | --- | --- |
| *LcSAIV1* | LITCHI022826 | acid β-fructofuranosidase 2 vacuolar | CTCAAGGTGGAAGAACAT | ATGGTAGTCTCAGTAGCA |
| *LcSAIV2* | LITCHI009111 | soluble acid invertase | GATACTGAATATGATGACT | AACTCTATACTTCTCTGT |
| *LcSNIV1* | LITCHI014297 | alkaline/neutral | CGTTACAAGACAGAAGAA | TAGGTTGCCGATAAGATA |
| *LcSNIV2* | LITCHI024492 | neutral invertase | TGATGATACTGCTACTGA | ACAATATAATCCACCACAAT |
| *LcSNIV3* | LITCHI027144 | neutral invertase | ATCGTTACAAGACTGAAG | CAATTAGATAGCCACCTT |
| *LcSNIV4* | LITCHI004126 | alkaline/neutral invertase | CTATCAACAACAGACTCAG | GGTATCCAATCCATTAGC |
| *LcPDC1* | LITCHI029074 | pyruvate decarboxylase | AGGTAACCACTCAGGATA | ACATTGTAAGGACCATCAT |
| *LcPDC2* | LITCHI018164 | pyruvate decarboxylase 2 | ATTCTTCACCACACTATT | GTTACAACCAATGCTAAT |
| *LcADH1* | LITCHI007374 | alcohol dehydrogenase 1B | GCTGAGTATTGTGTTGTG | ATAGCACCATAAGCAGTAA |
| *LcADHL1* | LITCHI003007 | alcohol dehydrogenase-like 3 | GAATTACAGATGGAATGG | TTGATTGGTATTGTTAGAC |
| *LcActin* | HQ615689(NCBI) |  | GGTAACATTGTGCTCAGTGGTGG | AACGACCTTAATCTTCATGCTGC |
